# Accuracy of electrocardiographic criteria for atrial enlargement: validation with cardiovascular magnetic resonance

**DOI:** 10.1186/1532-429X-10-7

**Published:** 2008-01-25

**Authors:** Connie W Tsao, Mark E Josephson, Thomas H Hauser, T David O'Halloran, Anupam Agarwal, Warren J Manning, Susan B Yeon

**Affiliations:** 1 Harvard-Thorndike Laboratory and the Department of Medicine, Cardiovascular Division, Beth Israel Deaconess Medical Center and Harvard Medical School, 330 Brookline Avenue, Boston, Massachusetts, USA; 2 Cardiovascular and Metabolic Division, GlaxoSmithKline Pharmaceuticals, 1250 Collegeville Road, Collegeville, Pennsylvania, USA; 3 Department of Radiology, Beth Israel Deaconess Medical Center and Harvard Medical School, 330 Brookline Avenue, Boston, Massachusetts, USA

## Abstract

**Background:**

Anatomic atrial enlargement is associated with significant morbidity and mortality. However, atrial enlargement may not correlate with clinical measures such as electrocardiographic (ECG) criteria. Past studies correlating ECG criteria with anatomic measures mainly used inferior M-mode or two-dimensional echocardiographic data. We sought to determine the accuracy of the ECG to predict anatomic atrial enlargement as determined by volumetric cardiovascular magnetic resonance (CMR).

**Methods:**

ECG criteria for left (LAE) and right atrial enlargement (RAE) were compared to CMR atrial volume index measurements for 275 consecutive subjects referred for CMR (67% males, 51 ± 14 years). ECG criteria for LAE and RAE were assessed by an expert observer blinded to CMR data. Atrial volume index was computed using the biplane area-length method.

**Results:**

The prevalence of CMR LAE and RAE was 28% and 11%, respectively, and by any ECG criteria was 82% and 5%, respectively. Though nonspecific, the presence of at least one ECG criteria for LAE was 90% sensitive for CMR LAE. The individual criteria P mitrale, P wave axis < 30°, and negative P terminal force in V1 (NPTF-V1) > 0.04s·mm were 88–99% specific although not sensitive for CMR LAE. ECG was insensitive but 96–100% specific for CMR RAE.

**Conclusion:**

The presence of at least one ECG criteria for LAE is sensitive but not specific for anatomic LAE. Individual criteria for LAE, including P mitrale, P wave axis < 30°, or NPTF-V1 > 0.04s·mm are highly specific, though not sensitive. ECG is highly specific but insensitive for RAE. Individual ECG P wave changes do not reliably both detect and predict anatomic atrial enlargement.

## Introduction

Atrial enlargement is a marker of increased cardiovascular events. Anatomic left atrial (LA) enlargement (LAE) is a marker of left ventricular (LV) diastolic dysfunction [1] and is associated with an abnormal stress test in subjects with known or suspected coronary artery disease [2]. In addition, it is a predictor for the development of atrial fibrillation [3-6], congestive heart failure [6], stroke [7], increased cardiac mortality [7,8], incidence and survival after myocardial infarction [9-11], and combined cardiovascular events [10,12]. Right atrial (RA) enlargement (RAE) is associated with increased risk for congestive heart failure [13] and increased mortality in patients with primary pulmonary hypertension [14].

The electrocardiogram (ECG) is used ubiquitously in clinical practice to evaluate patients with cardiac disease. ECG criteria for atrial enlargement have shown poor sensitivity and moderate specificity for detecting enlargement as defined by two-dimensional echocardiography parasternal long axis (PLAX) and four-chamber (4 Ch) measurements [15-23]. However, echocardiographic measures are subject to error due to variability in acquisition of appropriately aligned images, limitations in acoustic windows, and other causes of technical variation. In addition, because the LA is not a sphere with a constant radius [24], uni- or two- dimensional (2-D) echocardiographic measurements may not reflect true chamber size. Cardiovascular magnetic resonance (CMR) imaging can provide images in standardized planes, thereby providing more precise volumetric assessment of cardiac chamber size. We sought to determine the accuracy of the ECG for detection of atrial enlargement (AE) using the gold standard of volumetric CMR.

## Methods

### Study population

The study population consisted of 275 consecutive subjects referred for CMR either for clinical (n= 255) or research (n = 20) purposes, between February 2001 and July 2004. Clinical CMR indications included evaluation for the following: left ventricular function (n = 111), pulmonary veins (n = 74), coronary arteries (n = 47), perfusion and/or viability (n = 43), cardiomyopathy (n = 38), intracardiac shunt (n = 4), valvular function (n = 3), pericardium (n = 2), myocarditis (n = 1), and cardiac mass (n = 1). Many subjects were referred for more than one indication. Subjects were excluded if no sinus rhythm ECG was available (see below). Age, gender, height, weight, as well as history of atrial fibrillation (AF) and hypertension were recorded. Research subjects gave informed consent according to a protocol approved by the hospital institutional review board.

### Electrocardiography

A standard 12-lead ECG was performed on the same day of the CMR in all subjects in sinus rhythm (n = 201). An ECG in sinus rhythm, no more than 35 days from the CMR scan and prior to any pulmonary vein ablation procedures, was obtained for subjects with AF (n = 74). ECG LAE was defined by any one of the following: 1) P wave in any lead > 0.11s, 2) Notched P wave with interpeak duration > 0.04s (P mitrale), 3) P wave axis < 30°, 4) Area of negative P terminal force in lead V1 (NPTF-V1) > 0.04s·mm, or 5) Positive P terminal force in aVL (PPTF-aVL) > 0.5 mm [16,18,23,25]. ECG RAE included 1) P wave in inferior leads II, III, aVF > 2.5 mm or 2) Positive P wave in V1 > 1.5 mm [26,27]. All ECG determinations were made by one experienced observer (MEJ) blinded to other results.

### CMR technique

CMR was performed on a 1.5T whole-body scanner (Gyroscan NT, Philips Medical Systems, NL). Among subjects with AF, the CMR scan was performed prior to any pulmonary vein isolation procedure. Participants were imaged in the supine position with a phased-array five-element cardiac synergy coil for radiofrequency signal reception. Localizing scans were followed by free-breathing axial spin-echo (repetition time 800 ms, echo time 20 ms, field of view 300 mm, matrix size 192 × 512, slice thickness 6 mm, 0.5 mm gap) and end-expiratory, breath-hold, ECG-gated SSFP acquisitions (temporal resolution 33 ms, repetition time 3.1 ms, echo time 1.6 ms, flip angle 60 degrees, field of view 400 mm, matrix size 208 × 256, slice thickness 10 mm, gap = 0).

### Image analysis

**Figure 1:**
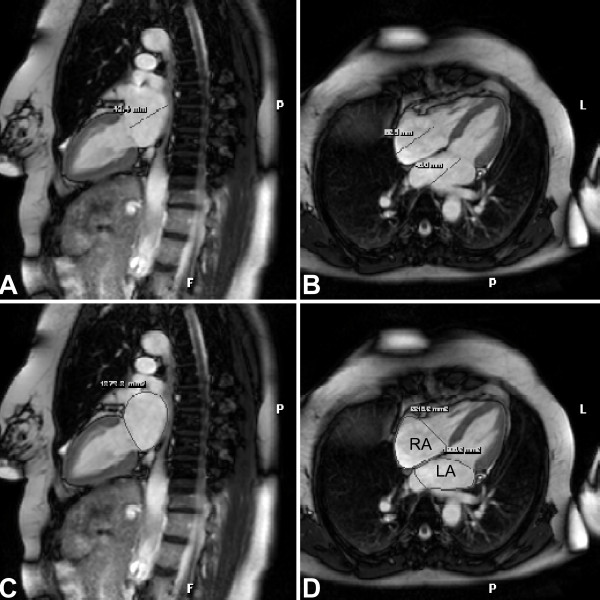
CMR images with representative measurements at atrial end-diastole. (A) Two-chamber (2 Ch) view with left atrial (LA) length measurement. (B) Apical four-chamber (4 Ch) view with LA and right atrial (RA) 4 Ch length measurement. (C) 2 Ch view with LA endocardial border tracings. (D) 4 Ch view with LA and RA endocardial border tracings. P: Posterior. L: Left.

CMR analyses were performed using dedicated software (EasyVision 5.1, Philips Medical Systems, Best, NL). LA length was measured from the posterior wall to the plane of the mitral annulus, parallel to the long-axis of the heart, in the two-chamber (2 Ch) and four-chamber (4 Ch) orientations on cine SSFP acquisitions at maximum atrial diastole (Figure 1A, B). Maximum atrial diastole was defined as the image immediately preceding the opening of the mitral and tricuspid valves. Similarly, RA length was measured from the posterior wall of the RA to the plane of the tricuspid annulus in the 4 Ch orientation (Figure 1B). LA area in apical 2 Ch and 4 Ch orientation were planimetered by tracing the endocardial border in maximum atrial diastole, excluding the confluence of the pulmonary veins and LA appendage (Figure 1C, D). Similarly, the 4 Ch RA area was planimetered, excluding the confluence of the vena cavae and the atrial appendage (Figure 1D). The borders of the atria were delimited at the planes of the AV annulus and the junctions of venous inflow. Atrial volumes were calculated according to the biplane area-length method, then indexed for body surface area (BSA) [28-30]. Enlarged CMR volume index was defined to be ≥ 55 ml/m^2^, two standard deviations (SD) above the mean of normal, healthy subjects in published population studies [31-36]. CMR analyses were performed by two experienced observers (CWT, TDO) blinded to other results.

### Statistical analysis

Continuous data are presented as mean ± SD. Categorical data are presented as counts and percentages. The difference in continuous measures between subject subgroups was assessed using Student's t test. The difference in categorical measures between subject subgroups and the prevalence of AE among the subject subgroups were assessed using Fisher's exact test. Sensitivity, specificity, positive predictive value (PPV) and negative predictive value (NPV) were used to assess the test performance for ECG LAE and RAE. The accuracy of ECG LAE and RAE were computed individually and in combination. The independent relationships of hypertension and CMR LAE to ECG LAE were further evaluated with logistic regression. A p-value of ≤ 0.05 was considered statistically significant. All statistical analyses were performed using SAS for Windows (v.9.1, SAS Institute, Cary, NC).

## Results

### Study population

**Figure 2:**
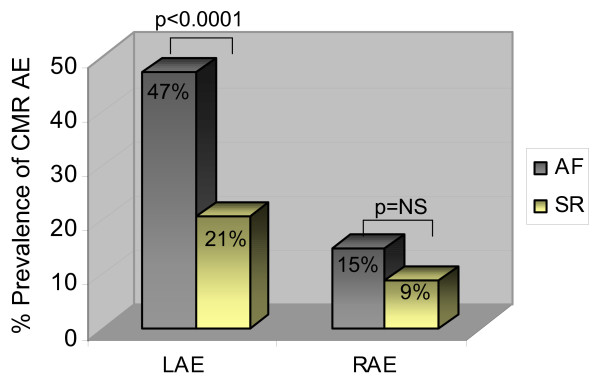
Prevalence of CMR atrial enlargement (AE) among subjects in sinus rhythm (SR) and with atrial fibrillation (AF). Subjects with AF have a significantly higher prevalence of left (LAE) but not right atrial enlargement (RAE), compared with subjects in SR.

The 275 study subjects consisted of 184 (67%) males, 91 females (age 51 ± 14 years; range 18 to 83 years) who were consecutively referred for clinical or research CMR study, including 27% subjects with a history of AF and 34% with a history of hypertension. For the entire group, CMR LA and RA volumes were 97 ± 38 ml and 79 ± 32 ml, respectively, and LA and RA volume indices were 49 ± 17 ml/m^2 ^and 40 ± 15 ml/m^2^, respectively. The prevalence of CMR AE and ECG AE is presented in Table 1. Normotensive subjects and those with a history of hypertension had a similar prevalence of CMR LAE (30%, p = 1.0). Compared to subjects in sinus rhythm, subjects with a history of AF had a significantly higher prevalence of CMR LAE and but not CMR RAE (Figure 2).

**Table 1:** Prevalence of Atrial Enlargement by CMR and ECG

**LAE**		**RAE**	
**CMR Criteria**			

**Volume index ≥ 55 ml/m^2^**	28%	**Volume index ≥ 55 ml/m^2^**	11%

**ECG Criteria**			

**Any ECG criteria for LAE**	82%	**Any ECG criteria for RAE**	5%
**P > 0.11s**	70%	**Inferior P waves > 2.5 mm**	1%
**P mitrale**	3%	**Positive P wave in V1 > 1.5 mm**	4%
**P axis < 30°**	9%		
**NPTF-V1 > 0.04s·mm**	16%		
**PPTF-aVL > 0.5 mm**	41%		

CMR: Cardiovascular magnetic resonance. ECG: Electrocardiography. LAE: Left atrial enlargement. RAE: Right atrial enlargement. P mitrale: Notched P wave with interpeak duration > 0.04s. NPTF-V1 > 0.04s·mm: Area of negative P terminal force in lead V1 > 0.04s·mm. PPTF-aVL: Positive P terminal force in lead aVL. Inferior P waves: P wave in any of the leads II, III, or aVF.

### Accuracy of ECG for anatomic atrial enlargement

The accuracy of ECG LAE is presented in Table 2. The presence of at least one ECG LAE criteria had a sensitivity of 90% with a NPV of 84% and specificity of 21% for detection of CMR LAE. When ECG criteria were analyzed independently, P wave > 0.11s offered the highest sensitivity for CMR LAE, with a NPV of 85%. Three individual ECG criteria were very specific for detecting CMR LAE. The presence of P mitrale, P wave axis < 30°, and NPTF-V1 > 0.04s·mm were 99%, 90%, and 88% specific, respectively, for detecting CMR LAE, although these criteria were not sensitive. The presence of P mitrale had a high PPV of 86% for CMR LAE.

**Table 2:** Accuracy of ECG criteria for Left and Right Atrial Enlargement as Detected by CMR

**ECG Criteria**	**Sensitivity (%)**	**Specificity (%)**	**PPV (%)**	**NPV (%)**
**LAE**				

**Any ECG Criteria for LAE**	90	21	31	84
**P > 0.11s**	84	35	34	85
**P mitrale**	8	99	86	74
**P axis < 30**°	8	90	23	71
**NPTF-V1 > 0.04s·mm**	37	88	47	76
**PPTF-aVL > 0.5 mm**	44	60	30	73

**RAE**				

**Any ECG Criteria for RAE**	17	96	33	91
**Inferior P waves > 2.5 mm**	7	100	67	90
**Positive P wave in V1 > 1.5 mm**	10	96	25	90

CMR: Cardiovascular magnetic resonance. ECG: Electrocardiography. PPV: Positive predictive value. NPV: Negative predictive value. LAE: Left atrial enlargement. RAE: Right atrial enlargement. P mitrale: Notched P wave with interpeak duration > 0.04s. NPTF-V1 > 0.04s·mm: Area of negative P terminal force in lead V1 > 0.04s·mm. PPTF-aVL: Positive P terminal force in lead aVL. Inferior P waves: P wave in any of the leads II, III, or aVF.

The ECG was insensitive but highly specific for CMR RAE (Table 2). The presence of P wave in leads II, III, or aVF > 2.5 mm or positive P wave in V1 > 1.5 mm had specificities of 100% and 96%, respectively, for diagnosing CMR RAE. Both criteria had a NPV of 90%, indicating low rates of false-negative results.

### Association of hypertension with ECG LAE

Because hypertension may affect P wave characteristics on ECG independently of atrial size [37], we performed logistic regression to assess the independent relationships of hypertension and CMR LAE to ECG LAE. With simultaneous adjustment, only CMR LAE, and not the presence of hypertension, was significantly associated with ECG LAE (OR 2.9, 95% CI 1.2 to 7.3, p = 0.023).

## Discussion

The commonly used ECG criteria for atrial enlargement have been compared to anatomic atrial size by M-mode or 2-D echocardiography [15-23,38], which have inherent disadvantages including limited acoustic windows and spatial resolution, geometric assumptions made for estimation of atrial size, and moderate interobserver variability of measurements. The goal of this study was to characterize the accuracy of commonly used ECG atrial enlargement criteria against a gold standard of volumetric CMR measurements.

The presence of any ECG LAE criteria was 90% sensitive with a NPV of 84%, but had poor specificity of only 21% for CMR LAE. Using a combination of ECG LAE criteria is thus highly sensitive for detection of anatomic LAE, with a low false negative rate. This study indicates that commonly used ECG LAE criteria are more sensitive for anatomic LAE than the 6–69% sensitivity previously reported using echocardiographic standards [16,18,22,23,38]. The criteria of P mitrale, P wave axis < 30°, and terminal P wave in V1 > 0.04s·mm were each highly specific although not sensitive for CMR LAE. In our CMR study, we found higher specificities of P mitrale and terminal P wave in V1 > 0.04s·mm for anatomic LAE than those obtained by similar atrial bi-plane volume analysis using 2-D echocardiography [38]. In addition, the high specificity of the criteria of P wave axis < 30° to detect LAE has not been previously described to our knowledge. Compared with prior M-mode echocardiographic studies [16,18,23,25], we found higher sensitivity but lower specificity for P > 0.11s, and similar excellent specificity for P mitrale and NPTF-V1 > 0.04s·mm to detect CMR LAE. The overall increased sensitivity of ECG LAE compared to ECG RAE may be explained by a larger number of criteria used, as well as high prevalence of the very sensitive criteria P > 0.11s.

Our results indicate that ECG criteria are insensitive but highly specific to detect CMR RAE, and are in agreement with analyses using echocardiographic volumetric RAE [39]. We found similar sensitivity but greatly improved specificity and both positive and negative predictive values of ECG for CMR RAE compared to 2-D echocardiographic standards [40]. No individual ECG criteria had both high sensitivity and specificity for predicting anatomic RAE. Though many of the commonly used individual ECG LAE and RAE criteria have high specificity for anatomic AE, their prevalence is rare even in our referral population.

Our CMR measurements are in agreement with Anderson et al [41], who reported higher CMR normative upper reference range atrial dimensions compared to echocardiographic standards. Though our mean volume indices exceed normative values by the American Society of Echocardiography [42], CMR volumetric measures have been shown to exceed that of echocardiography by 14–37% [41,43]. CMR measurements in this study are consistent with those obtained in past CMR studies [29,32-36], which published upper limits of normative LA volumes ranging 50–69 ml/m^2^. Within CMR studies, there is a range of volumes reported in healthy subjects, with smaller volumes found in studies with younger, as opposed to middle-aged, subjects [36,44,45].

Abnormalties in atrial conduction resulting from hypertension, elevated pulmonary capillary wedge pressure, or intrinsic conduction defects are associated with abnormalities in the P wave [37,46,47] and may be independent of atrial size. Thus, it is not surprising that in our study, ECG LAE criteria were sensitive but not specific. Since multiple etiologies may account for similar conduction disturbances in the P wave, the manifestation of such ECG criteria may be more accurately described as indicating atrial "abnormality" rather than "enlargement".

In logistic regression analysis, we demonstrated that CMR LAE was associated with ECG LAE independently of hypertension. However, a limitation of our study was that we were not able to adjust for other factors which may affect P wave morphology, such as the use of sodium channel blocking medications, as this information was unavailable. In addition, since this study was noninvasive, we were not able to measure atrial pressures, which may also affect the P wave [46].

## Conclusion

Our results further validate the strengths and weaknesses of commonly used ECG LAE and RAE criteria against the gold standard of anatomic measurements. Compared with volumetric CMR measures, none of the commonly used ECG LAE or RAE criteria provided high accuracy for detecting anatomic LAE or RAE. High sensitivity was achieved only with lower specificity and vice versa. The presence of any ECG LAE criteria and the individual criterion of P > 0.11s were highly sensitive for CMR LAE, while the criteria P mitrale, P wave axis < 30°, and NPTF-V1 > 0.04s·mm were very specific, though poorly sensitive, for detecting CMR LAE. ECG RAE criteria were highly specific, though insensitive, for detecting anatomic RAE. The limited specificity of the most sensitive ECG criteria for anatomic atrial enlargement is consistent with the fact that P wave perturbations reflect changes in atrial conduction, which may be due to a variety of etiologies. Thus, the term atrial "abnormality" may be more appropriate than "enlargement" to describe such ECG findings.

## Competing interests

The author(s) declare that they have no competing interests.
